# Proposal for a national diagnostics action plan for the United States

**DOI:** 10.1016/j.hpopen.2023.100099

**Published:** 2023-06-24

**Authors:** Gigi Kwik Gronvall, Sujeet B. Rao, Susan Van Meter, Adam Borden, Tom Inglesby

**Affiliations:** a Department of Environmental Health and Engineering, Johns Hopkins Bloomberg School of Public Health, United States; b American Clinical Laboratory Association, United States; c Johns Hopkins Center for Health Security, United States; d Independed scholar

**Keywords:** Pandemic preparedness, Diagnostic testing, COVID-19 response, Mpox, Laboratory testing

## Abstract

Providing a definitive diagnostic test in a disease emergency is critical to limit pathogen spread, develop and deploy medical countermeasures, and mitigate the social and economic harms of a serious epidemic. While major accomplishments have accelerated test development, expanded laboratory testing capacity, and established widespread point-of-care testing, the United States does not have a plan to rapidly respond, to develop, manufacture, deploy, and sustain diagnostic testing at a national scale. To address this gap, we are proposing a **National Diagnostics Action Plan** that describes the steps that are urgently needed to prepare for future infectious disease emergencies, as well as the actions we must take at the first signs of such’ events. These recommendations require substantial collaboration between the US government (USG) and the private sector to solve a series of challenges now, as well as to prepare for the massive and rapid scale-up of laboratory and point-of-care test development and testing capacity in future emergencies. The recommendations include establishing pre-event contracts; ensuring rapid access to clinical samples; creating a permanent public–private testing coordinating body to allow for rapid information sharing and improved cooperation among the USG, test developers, and clinical laboratories; and accelerating testing rollout at the beginning of an event—and thus, the effective public health management of a disease crisis.

## Highlights

• A definitive diagnostic test for an infectious disease is critical for limiting disease spread, developing and deploying medical countermeasures, and mitigating social and economic harms.
• The US government should facilitate contracts with testing manufacturers and laboratories before a disease emergency, so testing can be rapidly scaled up.
• A permanent public-private testing coordinating body can facilitate information sharing, clinical sample access, and improved cooperation.
• Early in a disease emergency, the US should swiftly establish billing codes, widespread coverage, and national payment rates for new tests.
• The diagnostic testing ecosystem is part of the nation’s critical infrastructure and should receive support pre-event and during a response effort.

## 1 Introduction

Providing a definitive diagnostic test for an emerging infectious disease as rapidly as possible is critical for limiting disease spread, developing and deploying medical countermeasures, and mitigating the social and economic harms of a disease emergency. During the COVID-19 and mpox responses, extraordinary expertise within and outside of government led to major accomplishments in the acceleration of test development, expansion of laboratory testing capacity, and deployment of widespread point-of-care testing. But these processes took substantial time to put in place and have not yet been institutionalized. Developing a plan and a robust system to rapidly develop, manufacture, and implement nationwide diagnostic testing in the earliest days of a new infectious disease crisis is of critical importance. Insufficiencies in diagnostic testing during the early days of the COVID-19 pandemic and the mpox epidemic—and the consequences of those gaps—are well-documented and demonstrate the need for the US government (USG) to have a plan for diagnostic testing to support future emergency public health responses.

Scaling up diagnostic testing to the levels required nationwide necessitates substantial work with the private sector, both to prepare for and to immediately respond at the start of a crisis. The value of the private sector in a national diagnostics response was demonstrated repeatedly during the COVID-19 pandemic emergency, during which public–private coordination led to an unprecedented mobilization of private-sector laboratory-based and point-of-care test development and expanded laboratory testing capacity. The private sector’s value was also demonstrated during the 2022 mpox epidemic response, when the USG collaborated with commercial laboratories to expand national testing capacity. By early July 2022, five commercial laboratories were partnered with the Centers for Disease Control and Prevention (CDC), augmenting the nation’s mpox testing capacity to 80,000 tests per week—beyond the 10,000 tests per week the agency’s Laboratory Response Network (LRN) could provide on its own—and easing the workflow required of clinicians to submit samples for testing. [2] This additional capacity ensured that at the height of the mpox epidemic in August 2022, when 18,500 patient samples were being tested weekly with the potential for additional spread, testing volume was robust. [3] During the COVID-19 and mpox responses, the USG formed collaborations and contracts with private-sector test manufacturers and commercial clinical laboratories, supported the expansion of manufacturing and laboratory capacity, and assisted in the development and validation of tests. For example, the National Institutes of Health (NIH) created the Rapid Acceleration of Diagnostics (RADx) initiative to speed innovation and commercialization of laboratory-based, point-of-care, and home- based diagnostics for COVID-19 and to make testing widely available to the public, particularly for vulnerable and disproportionately affected populations. [4] One RADx effort, the Independent Test Assessment Program (ITAP)—initially designed to accelerate regulatory review by the Food and Drug Administration (FDA) of COVID-19 over-the-counter tests—later expanded to review mpox tests. ITAP is now a well-regarded model of how swift public–private collaboration can hasten the pace of innovative test development and ready tests for FDA consideration. [5] Notably, the FDA’s implementation of frequent town hall meetings with test developers and distribution of submission templates usefully guided private sector test developers seeking FDA authorization and expedited that process. [6]

Now is the time to evaluate the mechanisms developed during the pandemic—and expanded upon the subsequent mpox emergency—to scale up diagnostic testing, determine what processes should be retained or expanded, and develop a durable plan to reduce lags and minimize gaps in the national approach to diagnostic testing during future disease emergencies. To inform our recommendations for such a plan, we conducted informative conversations with 20 experts from the USG and the private sector who played crucial roles in the scale-up of diagnostic testing for COVID-19 and mpox {Appendix A]. These discussions illuminated areas where additional policy and contracting mechanisms, regulatory flexibilities, incentives, requirements, or funding could streamline the development and scaling of diagnostic testing capacity to accelerate clinical and public access and improve the public health response. We reviewed the literature regarding the challenges and successes of diagnostic testing for COVID-19, monkeypox [1], Mpox14), and Zika, along with government agency and independent assessments of the US testing capacity during past pandemic and epidemic events. We examined technology trends that may affect diagnostics scale-up in the future, such as CRISPR-based tools. [7] We also benefited from author experiences with different vantage points of diagnostic testing during the COVID-19 and mpox emergencies: as part of the White House COVID-19 response, with the medical technology trade association AdvaMed, the national clinical laboratory association, the American Clinical Laboratory Association, and in developing the Johns Hopkins COVID-19 Testing Toolkit. [8], [9].

## 2 Challenges to developing testing capacity during COVID-19

In the earliest days of the COVID-19 pandemic, before evidence of domestic US transmission emerged, some diagnostics developers waited for clear signals that a testing market would materialize, having previously developed tests for outbreaks, such as H1N1 (2009), where demand was negligible and payment modest. Eventually, demand for testing rose to unprecedented levels, requiring the mobilization of public- private collaborations and the execution of scores of agreements. Those agreements— including those to build out manufacturing and laboratory testing capacity—were sometimes developed hastily, often applied only short-term, and failed to eliminate market uncertainty to secure robust capacity through upturns and downturns in demand.

Typically, a complex set of factors influences private-sector test developers’ decisions to develop a new diagnostic test, ramp up manufacturing, pivot existing manufacturing lines, and/or scale up laboratory testing capacity. These factors include the projected scale and type of outbreak, which may determine eventual test demand and market size; the availability of patient samples needed to develop and validate tests; the availability of supplies needed for developing tests, including test controls, probes, primers, reagents, and precision plastics; and the accessibility of other necessities for test sample collection and transport, like swabs. Companies also weigh variables such as regulatory pathways needed to bring a test to market and the timing and character of billing codes, insurance coverage, and payment policies. Arranging the workflow of test development and scale-up combines a complicated series of steps; therefore, delays in the availability of samples, supplies, and components can set back critical decision-making and may be enough to prevent testing capabilities from coming online in a useful timeframe.

### 2.1 Financial decision-making

The economics and financial decision-making surrounding diagnostic test development or production scale-up also are complex. During COVID-19, the USG took actions to positively influence the private sector to develop tests and ramp up laboratory testing capacity; for example, the government took steps to “de-risk” the market for test developers, by making investments to expand manufacturing capacity through the Department of Defense or issuing procurement contracts through the HHS Office of the Assistant Secretary for Preparedness and Response (ASPR).

Additional government actions included the passage of federal legislation establishing comprehensive coverage requirements and policies for Medicare, Medicaid, and commercial insurers [10], [11] The Centers for Medicare & Medicaid Services (CMS) enhanced reimbursement rates for high-throughput molecular laboratory testing, which helped support the expansion of laboratory capacity and bolster commercial laboratory logistical operations transporting patient samples across the country. Processes for establishing widespread coverage without cost-sharing for all types of COVID-19 tests, realizing the goal of the CARES Act, were cumbersome, and there were limits placed on funding to support testing for uninsured individuals, creating significant challenges for equitable access to testing when that funding ran out. The CDC gave clinical guidance to providers and the public about use cases for various screening and diagnostic tests, and there were significant US efforts to engage with private-sector test developers to facilitate testing access expansion and address problems that arose.

### 2.2 Regulatory pathways

There were additional lessons learned in the diagnostic testing scale-up for COVID-19, such as the importance of ensuring regulatory certainty for all test developers, patients, and public health officials in ways that did not compromise access to early testing. The declaration of a Public Health Emergency (PHE) in February 2020 allowed the FDA to initiate an Emergency Use Authorization (EUA) process, requiring all test developers to seek EUA and allowing the agency to authorize diagnostic tests for use during the COVID-19 emergency.

The EUA regulatory requirements helped commercial manufacturers enter the market, but, as has been well-documented, they initially complicated and slowed the use of early laboratory developed tests (LDTs)—a type of *in vitro* diagnostic test that is designed, manufactured, and used within a single laboratory and most frequently the first types of tests available in an emergency. [12] Over time, the FDA allowed for a process in which laboratories could notify the agency of an LDT and had 15 days to prepare an EUA submission to the agency. The implementation of FDA town hall meetings for test developers and templates to guide submissions improved the process. For future emergencies, an EUA process that does not restrict the use of LDTs can be established.

Challenges also were presented when exceptions to EUA were removed, as was the case temporarily in 2020 for point-of-care antibody/serology tests. At that time, the lifting of regulatory review flooded the US market with point-of-care serology tests that yielded innacurate results, undermining patient and public health confidence in testing. [13] Once reinstated, the NIH supported the review of serology tests to EUA standards.

### 2.3 Contracting and reporting arrangements

The USG entered many contracts and arrangements related to COVID-19 diagnostic testing, accomplishing a great deal in partnership with the private sector. Notably, however, there were often lags of several or more weeks between identifying the need to boost manufacturing or testing capacity and executing contracts to expand that capacity. The demand from the COVID-19 Delta and Omicron surges exceeded the nation’s existing testing capacity, requiring the USG and private-sector partners to move urgently to meet testing needs.

The lack of a “one-stop shop” for the private sector to engage the USG hindered the development of tests and testing capacity, especially when there was a lack of clarity about what the government wanted and needed for testing. The private sector also found test-reporting requirements challenging, as federal, state, and municipal requirements were often inconsistent, and laboratories were frequently made to provide patient demographic data often inaccessible to them in the normal course of operations.

## 3 Developing a plan to scale up testing in a future disease emergency

The next time there is an infectious disease emergency, diagnostic test development and scale-up should be informed by a predetermined, agreed upon plan, not an ad hoc, bespoke process. A plan for developing and deploying testing enhances the public health response by allowing definitive diagnoses and situational awareness, even if many details about the character, durability, or severity of the epidemic are unknown in the beginning. The capability to deploy large-scale testing should be thought of as calling the fire department to the scene of a fire; a quick, overwhelming, and strong response could limit the size of the incident.

To that end, during the fall of 2022 we developed a set of recommendations for providing rapid and enduring testing capacity in the US during the next infectious disease emergency, sharingthe recommendations with policy makers as a preprint- while this paper was under review. Most of the recommendations need to be implemented in the short- to mid-term, well ahead of any future infectious disease crisis, to be available to inform the next rapid—and potentially nationwide—emergency response.

Since we developed these recommendations, the PREVENT Pandemics Act was passed in December of 2022 as part of the Consolidated Appropriations Act of 2023^1^. Some of the recommendations in this Proposal for a National Diagnostics Action Plan were included, in whole, or in part, in the new law. While these steps are important for improving the nation’s preparedness, there is still work to be done.

1. Establish a permanent public–private National Testing Coordination Forum focused on preparedness and response to disease emergencies.

Currently, jurisdiction of aspects of responsibilities for diagnostic testing is are dispersed throughout the USG from White House-drive response efforts to various agencies within the Department of Health and Human Services (HHS), including CDC, CMS, FDA, and NIH. This leads to, promoting challenges for efficient efficiency, and effective intra-governmental collaboration and coordination, and collaboration and makes it difficult for and certainly difficulties for private sector test developers to fully discern signals and policy from USG that could assist in the private sector’s preparedness and response to a future emergency. Regularizing this coordination and collaboration, specific to for testing is essential. Under an Executive Order, or via legislative authorization, the USG should develop a permanent and durable National Testing Coordination Forum (or federal advisory committee) within the Office of the Secretary of the Department of Health and Human Services (HHS). This Forum could be part of the Public Health Emergency Medical Countermeasures Enterprise (PHEMCE). Notably, a National Academies committee tasked with recommending improvements in public health responses to disease emergencies recommended the development of such an advisory board for the PHEMCE. [14] The PREVENT Act did not include policy to establish this Forum.

The Forum would meet regularly and be empowered to provide recommendations to the HHS Secretary, White House, and Congress on both preparedness and response matters specific to diagnostic testing. During the COVID-19 pandemic, an Executive Order in early 2021 established a narrow and temporary public sector-only testing board. [15] We recommend a more robust, public–private coordinating body. The Forum would play a critical role in the event of a disease emergency.

The Forum would comprise leaders from public health-sector agencies and departments integral to diagnostic testing (e.g., CDC, CMS, FDA, NIH), and representatives of public-sector laboratories, hospital laboratories, commercial laboratories, private- sector organizations that represent diagnostic manufacturers, and healthcare product distributors. Private-sector Forum participants should be representatives of industry trade organizations whose members have the capacity to rapidly develop, manufacture, or perform tests for a nationwide response and distributors with sophisticated supply operations.

The National Testing Coordination Forum would ensure that the USG works closely with the private sector to establish the partnership elements needed to scale up and sustain national testing capacity in a crisis. Further, the Forum would advise HHS on the need for the development of new diagnostic tests for diseases determined to be emerging public health threats on regional or national levels.

The Forum would serve as the primary coordinating hub for public- and private- sector stakeholders executing diagnostic testing plans to ensure real-time information sharing, swift development and scale-up of tests and testing capacity, and clear communications to the public on the state of testing. Recommendations would include use cases for various tests across all modalities, such as laboratory and point-of-care, including at-home and over-the-counter tests. Challenges seen in COVID-19 and other disease emergencies, such as access to pathogen samples needed for diagnostic test development or refinement, would be coordinated through this board.

The Forum also would identify serious national testing challenges and provide recommendations for solving them. For example, at the time of writing, clinical laboratories and manufacturers are preparing for potential cases of Ebola in the US, originating from the current outbreak in Uganda. Clinical laboratories need direction on decontaminating laboratory instruments if used for routine testing for patients suspected of having Ebolavirus. These instruments are complex, sensitive, and costly platforms that could be damaged with inappropriate decontamination efforts. While industry previously requested clarity on these issues (first in 2014), industry-friendly guidance has not yet been developed. Currently, multiple HHS agencies are working to develop such a protocol, in collaboration with manufacturers. The Forum, had such a body been created in 2014 when Ebola spread in West Africa and led to some US cases, could have directed the development of such a protocol then, leaving us better prepared today.

There is precedent for the development of such a body. Former President George W. Bush signed an Executive Order establishing the Office of the National Coordinator for Health Information Technology (ONC) to advance the adoption of electronic health records. Under the order, the new National Coordinator was directed to “coordinate outreach and consultation by the relevant executive branch agencies (including Federal commissions) with public and private parties of interest, including consumers, providers, payers, and administrators.” [16].

The Forum’s heightened, regular, data-driven coordination would allow for improved alignment of supply and demand in times of emergency and for the informed development of long-term policy to sustain bolstered manufacturing and laboratory capacity, even as demand drops in between spikes of increased testing needs. The Forum and its recommendations would facilitate preparedness for future emergencies, and its capabilities could be exercised in public health preparedness activities.

2. Facilitate transparent, bilateral contracts between the USG and testing manufacturers and laboratories before a disease emergency.

The USG should establish a series of contractual agreements with a core group of diagnostic manufacturers with a proven capacity to develop and manufacture components, supplies, and test kits at scale. The USG should also establish contractual agreements with commercial laboratories that have national reach and demonstrated expertise in developing tests, facilitating patient sample collection and transport, maintaining an expert workforce to perform tests, and efficiently delivering results at national scale. Testing capabilities should be considered part of the nation’s critical infrastructure.

The process would start with a request for information (RFI) seeking input regarding the most essential agreements. Based on information gathered through the RFIs, the USG would solicit requests for proposals (RFPs) from test manufacturers and clinical laboratories to fulfill a discrete number of bilateral project agreements. These contractual arrangements would be established prior to a crisis so that all critical components of the private-sector testing system are ready to respond immediately when needed.

Commercial manufacturers of diagnostic testing supplies and test kits could reserve manufacturing capacity for molecular and antigen testing, so that once triggered, manufacturers could dedicate agreed upon capacity to manufacture new or existing tests for a sustained period. Manufacturing capacity readiness should be established so that ramp- up time to pandemic peak supply demands can be reached within ∼ 30 days. Contracts could include procurement agreements, such as has been the case for at-home tests, to allow for continuous demand signals to support manufacturing capacity maintenance.

Commercial laboratories could reserve laboratory services to fulfill surge capabilities, from test development support to sample collection and transport, as well as capacity to ramp up and sustain an agreed upon level of weekly testing within ∼ 30 days, with targeted turnaround times to results reporting. For example, CDC has reached agreements with five commercial laboratories to hold capacity to run 10,000 mpox tests per month for the next several months, in the event it is needed.

During infectious disease emergencies, contracts with clinical laboratories should support the attraction, retention, and training of laboratory scientists, technicians, and other laboratory professionals in short supply. Clinical laboratories cannot perform tests without the expertise of laboratory staff. Staff shortages inherently limit laboratory capacity, particularly during public health emergencies.

South Korea used this pre-event, contractual agreement model to great effect in COVID-19, making molecular testing, including polymerase chain reaction (PCR), widely available, along with effective contract tracing, months earlier than they were available in the US. [17].

The PREVENT law includes new authorities for domestic warm base manufacturing capacity for medical countermeasures, directing BARDA to support the establishment and maintenance of such surge capacity and capabilities. The language is most focused on manufacturing and should be modified to clearly specify that clinical laboratory reserve capacity could also be effectively kept warm. Questions remain about whether HHS has a sufficient numbers of contracting experts to manage such agreements. When a Memorandum of Understanding with the Department of Defense for contracting services expires at the end of 2023 the specialized staffing shortages will be felt more acutely.

3. Bolster the US Strategic National Stockpile via vendor-managed inventory of critical diagnostic test components, materials, and final products.

The National Academies and others have recommended HHS reexamine Strategic National Stockpile (SNS) management in response to the shortcomings seen during the COVID-19 pandemic, particularly for materials needed for public health emergencies. [14] Specific to diagnostic testing, the USG should form agreements with commercial manufacturers to procure needed testing supplies as part of the SNS, or via Vendor Managed Inventory (VMI), in which manufacturers hold supplies onsite, rotating stock to remove expiring products. Critical testing supplies include pre-analytic supplies such as sample collection and transport media, as well as extraction reagent, probes, primers, test kits for known pathogens, precision plastics, and other critical supplies that were often in short supply during the pandemic.

Processes need to be implemented so that supplies from the SNS/VMI could be transferred directly to clinical laboratories in need. In the past, several instances saw supplies transferred to states and localities, which then needed to transfer the supplies to clinical laboratories, creating complexities, delays, and inefficiencies. Such agreements should also allow for a six-month supply of these components/materials/ test kits to either be kept onsite at commercial laboratories with national reach or dedicated to these laboratories but held by manufacturers for swift shipment to laboratories.

The PREVENT Actimproves the authorities that govern the SNS and allows for VMI to most efficiently and effectively leverage the SNS. The SNS can indeed include diagnostic testing supplies, though further legislative clarity could be useful as well as continued oversight by Congres to ensure that HHS is building out a robust SNS that includes diagnostics supplies. The PREVENT also requires an annual review of the SNS that includes an examination of supply chain vulnerabilities for products the SNS plans to purchase in the period covered by the review.

For a robust effort to create pre-event contracts, legislative action likely is needed. However, existing authorities under Title III of the Defense Production Act of 1950 that can support industrial base/technology issues might be able to be leveraged to address this need, as was done during the COVID-19 pandemic.

4. Standardize data reporting requirements and state reporting formats.

Robust data collection and analysis are essential to health emergency management and advancing equity, including by understanding test results and patient data that illuminate health disparities in infection and mortality rates. The USG should work to standardize data to improve national visibility and understanding about capacity distribution, trends, and gaps (whether regional or demographically based).

Today’s local, tribal, state, federal, and private health information exchange reporting requirements are a patchwork system that generates inefficient and unnecessarily duplicative health data, as well as costs to reporting providers. The USG should facilitate the establishment of a uniform, single, national, accurate, actionable public health data reporting policy that standardizes data sets and delivery mechanisms for data. Under such a system, appropriate entities would report public health data to CDC/HHS depending on their access to and control over the requested data. Then CDC/HHS could make the data available to state and local authorities simultaneously, removing the burden on states and localities to establish separate requirements and mechanisms. This streamlined system would improve access to accurate and timely data to inform the nation’s response efforts while also relieving the burdens created under today’s patchwork system.

Legislation is needed to improve data collection and reporting processes.

5. Invest in diagnostic testing research and development, while extending the reach of testing.

Investments in testing research and development will drive the next generation of screening and diagnostic tools across laboratory-based and point-of-care testing modalities and are needed now to prepare for the future. The development of at- home rapid testing for COVID-19 showed the great potential for innovation and novel approaches to testing in a national epidemic, but it also highlighted the current technical sensitivity limits of those technologies. Public-private sector collaborations could incentivize diagnostic testing manufacturers, component manufacturers, and laboratories to invest in innovation and development in the pre-analytic phase of testing, including for sample collection, stabilization, and transport. Such collaborations could also incentivize innovation in key testing components, such as antibodies for the development of antigen tests, extraction reagent for molecular testing, automated instrumentation, and new technologies to improve the speed and accuracy of testing for instrumented and instrumentless testing. Further, existing efforts to improve the accessibility of testing to individuals with disabilities should be augmented to further promote equity and ensure the availability of innovative tests to all.

HHS ASPR, through its Biomedical Advanced Research and Development Authority (BARDA), has provided significant support to accelerate the development of emergency diagnostic tests, as has the NIH RADx initiative, as described above.

Providing increased investment for these entities and programs and expanding their scope to allow them to focus on all infectious pathogens—including mycotics now largely absent from these programs—are essential actions to prepare for the next emergency.

Further, extending the reach of new technologies is critical to effective resopnse efforts. The CDC’s Increasing Community Access to Testing (ICATT) program, established during the pandemic to bring testing services to uninsured and at- risk populations and surge testing to state and local jurisdictions, should be made permanent. Over 10,000 rural and urban program sites were established in partnership with pharmacies expanded access to sample collection for laboratory-based and rapid point- of-care testing. This model allows for services to be brought directly to communities most heavily impacted by COVID-19. It should be carried forward to support testing and ultimately treatment, in collaboration with ASPR’s Test to Treat program, for additional pathogens. For example, the Biden administration’s focus on eliminating hepatitis C could be bolstered by leveraging this infrastructure. ICATT should be part of our nation’s preparedness and response.

The PREVENT Act, now law, created new authority for HHS to award grants, contracts, or cooperative agreements to entities, including laboratories, to specifically advance genomic sequencing of pathogens. Further, PREVENT added section 319B of the Public Health Service Act “ which allows the Secretary to “contract with public and private entities, as appropriate, to increase capacity in the rapid development, validation, manufacture, and dissemination of diagnostic tests, as appropriate, to State, local, and Tribal health departments and other appropriate entities for immediate public health response activities to address an emerging infectious disease with respect to which a public health emergency is declared under section 319, or that has significant potential to cause such a public health emergency.” Further, now established in the law is the Advanced Research Projects Agency-H, or ARPA-H, which may award grants or contracts to develop innovative new technologies for detecting pathogens.

Significant augmentation of BARDA, NIH RADxt, and authorization of ITAP programs would require legislation.

6. Increase support for USG programs dedicated to testing preparedness and response.

USG programs run by ASPR, FEMA, CDC, NIH, and FDA were critical to scaling up national testing capacity and developing public–private partnerships during the COVID-19 and mpox emergencies. These USG programs helped keep industry informed, answered questions, discovered company-specific information about testing production and manufacturing, and developed a testing demand model so that shortfalls/lack of availability in testing could be identified and mitigated. One program, the ASPR Industrial Base Expansion (IBx) effort, provided a portal for industry to engage with the government and find solutions to complex problems; for example, when a necessary ingredient is stuck on a ship in port, when a key scientist is experiencing delays getting their visa to visit a production facility, or if engagement with USDA is needed for antibody production. Going forward, this office should also look at supply chain management (for example, if several companies are sourcing an important resource from the same country or company, this poses risks). In addition, information-sharing mechanisms, such as the Diagnostic Evidence Accelerator, were important during COVID-19 and should be supported for future disease emergencies.

(20) Having more streamlined and coordinated action and communication between the USG and industry would be highly valuable to industry, and that may argue for consolidation of some of these activities into few program offices. But given that the research, development, regulation, contracting and clinical guidance around testing are quite distinct functions, it may be the case that testing activities need to reside in this array of programs for the longer run. In that case, coordination and communication among them and with industry will be crucial.

7. Plan to rapidly provide clinical samples to private-sector test developers during the earliest phase of a new epidemic and plan to quickly share clinical research to inform test development.

Early in the COVID-19 pandemic and during past newly emerging epidemics, delays in accessing patient samples slowed testing development. Samples are needed to develop and validate specific tests, and, importantly, they are critical for determining what type of test is most useful to limit disease spread, ideally providing a definitive diagnosis before an infected person is symptomatic. Additionally, knowledge that informs test development, such as the latest clinical research and test use cases, is often slow to be shared. The above discussed National Testing Coordination Forum could be a vehicle for public–private dissemination of patient samples and sharing of developing clinical research.

In the earliest stages of an emergency, when access to patient samples is limited, the Forum would support the work of CDC, FDA, and NIH to facilitate high-value allocation of samples to test developers under contract with the USG to develop and scale up manufacturing and testing capacity. Use of available sequencing, contrived, and spiked samples should be leveraged, as appropriate. The need to rapidly obtain clinical samples speaks to the importance of US global partnerships with the World Health Organization (WHO) and other public and private entities. The USG, working closely with the Forum, should be prepared to share the latest surveillance information and latest clinical samples with private-sector partners to ensure their efforts are tracking with any new and evolving epidemic.

Similarly, the USG, working with the Forum, should ensure the sharing of clinical research findings relevant to developing diagnostics for new pathogens of concern, in collaboration with CDC, providers, test developers, public health offices, and others. The dissemination of often rapidly evolving scientific and clinical understanding of a new pathogen can support the development of testing use cases and inform clinical trial design for diagnostic tests, all of which provides predictability for manufacturers and speeds up the process to provide quality testing.

The PREVENT, now law, did include a provision to require HHS to make public policies related to public and private entities accessing specimens of pathogens to support research and development of medical countermeasures, such as tests. This provision bolstered the Public Health Service Act with a new section, 319B, to allow HHS to contract with public and private entities to “increase capacity in the rapid development, validation, manufacture, and dissemination of diagnostic tests … to address an emerging infectious disease” that has caused, or has significant potential to cause, a PHE.

8. Refine regulatory requirements for Pre-EUA and EUA processes to swiftly bring testing to scale.

Regulatory certainty for all test developers, patients, and public health officials that facilitates early access to quality testing, at scale, is essential. Establishing a Pre-EUA program and leveraging EUA processes can provide swift access to validated testing in future emergencies. Ideally, at the beginning of a potential emergency, when a pathogen of concern is identified, the pre-emergency bilateral response contracts with test developers that the USG had in place with select test developers would be triggered. These agreements would allow test developers covered by them to access patient samples and swiftly develop and launch tests for patient care in a Pre-EUA environment. That is, laboratory developed tests (LDTs) should be leveraged in clinical setting during this period of time. Companies developing and manufacturing tests for the commercial market under pre-established bilateral agreements should be permitted Pre-EUA development status, to be established by the FDA. A limited number of commercial manufacturers with widespread placement of high-throughput platforms in commercial and other laboratories can achieve significant testing scale quickly. This approach can also protect FDA resources, allowing reviewers to focus on tests that can deliver the highest value to the nation’s response efforts.

Test developer experience during this period would assist with the establishment of test validation in the event of a PHE declaration and subsequent initiation of the FDA EUA process. If EUA is established, FDA should have the flexibility to apply the EUA to all test developers, as it did during the COVID-19 PHE, or to require notification only from LDT developers and EUA from manufacturers, as is being done during the mpox PHE.

For both Pre-EUA and EUA circumstances, FDA should leverage the work of test developers who have existing bilateral agreements to assist in the development of templates to streamline EUA submissions to the agency should the EUA requirement be instituted. Should EUA be required, FDA should allow test developers to provide notification of use of a validated test and a forthcoming submission, allowing 15 days to prepare the submission. During this stretch of time, tests could be used and pre- positioned at laboratories. Further, the agency should exercise regular test developer town hall meetings, again, as was done during COVID-19, to support test developers in preparing or updating submissions, as more is learned about a pathogen.

9. Swiftly establish billing codes, widespread coverage, and appropriate national payment rates for new tests.

To ensure robust provider and patient access to tests across all modalities (laboratory- based and point-of-care, including at-home and over-the-counter tests), the rapid establishment of medical billing codes, coverage, and national payment rates is essential. While expedited processes for coding are established, the US lacks durable policy to rapidly develop comprehensive coverage and payment to private-sector testing partners.

The American Medical Association Current Procedural Terminology (CPT®) Editorial Panel established a rapid process for the creation of new CPT codes during the COVID-19 pandemic. This now durable process was leveraged by public- and private- sector payers, providers, and other healthcare suppliers to accurately process claims for COVID-19-related services. The process also has been used successfully during the mpox PHE.

Importantly, at the outset of the COVID-19 pandemic, Congress took action to require coverage of COVID-19 tests by public and private insurers without cost-sharing, but the process was not swift, cannot be extended to other pathogens, and did not address payment rates. CMS initially relied upon typical processes that allowed each of the seven Medicare Administrative Contractors (MACs) to establish their own payment rate. Ultimately, CMS did enhance rates for high-throughput molecular laboratory testing in an effort to incentivize the expansion of laboratory test capacity and improve test result turnaround time. This augmentation of the payment rates was extraordinarily important to increasing capacity, eliminating reimbursement uncertainty, and dramatically extending the reach of testing. It is an approach worth replicating. As the AMA has established an expedited coding process, so too, CMS should develop a mechanism to set and communicate broad, national coverage and payment for testing of new pathogens of concern. Requirements for private insurers to establish such processes may best be accomplished via legislation.

10. Communicate clearly with healthcare workers and the public regarding diagnostic test use, goals, and interpretation.

Communications are as vital as testing capacity in helping ensure diagnostic test use supports a robust public health response to an emerging or new pathogen threat.

Diagnostic tests, including molecular, antigen, and serology tests, have different use cases,goals, and limitations that can shift over time based on a wide range of factors—particularly as knowledge about a new pathogen is gained. This changing context makes it far more likely that confusion or conflicting understandings will emerge among the public. Accounting for that, it is vital that all expert stakeholders—especially the USG—speak clearly and frequently about the role of testing at any given moment during a potential crisis.

For example, the struggle during COVID-19 to clearly articulate the screening and diagnostic use cases of rapid antigen tests may have undermined their use. If tests are scarce when a threat first emerges, the rationale for allocation and prioritization should be clearly articulated. If circumstances change, such that tests become much more widespread or the nature of a threat changes in a way that requires a different strategic approach to testing, officials should quickly and clearly communicate any new guidance and the underlying rationale. If different types of tests are developed with different use cases, officials should clearly communicate those specifics to the public to promote appropriate use.

Frequent and clear communication with industry is also needed, specifically about testing capacity needs and the current role of testing, such as whether testing is designed to diagnose suspected illness, identify possible areas of community spread, or be used for broader surveillance.

11. Include the nation’s diagnostics infrastructure as part of its critical infrastructure.

The COVID-19 and mpox PHEs have incontrovertibly shown that the American diagnostic testing ecosystem is part of the nation’s critical infrastructure and should receive support for the good of public health, both pre-event and during a response effort.

To ensure the health of the nation’s diagnostics infrastructure, policymakers should take steps via legislation to provide for long-term sustainable and predictable reimbursement to clinical laboratories. Mitigating the pending January 1, 2023, reductions of up to 15% for 800+ tests under the Medicare clinical laboratory fee schedule would provide for great predictability in payment for all test developers. Congress is currently contemplating bipartisan, bicameral legislation to mitigate the 2023 and subsequent reductions under current law. Predictable and sustainable Medicare payments support patient access, innovation, and clinical laboratory infrastructure.

Further, policymakers should advance administrative policies and legislation to incentivize the next generation of laboratory professionals to train in the field. Clinical laboratories across the country are facing shortages of laboratory scientists and technicians with few programs to train them. Proposals that would allow for federal support to expand existing training programs and loan forgiveness and repayment plans, as have been put in place for nurses and doctors, should be considered.

## 4 Conclusion

The American diagnostics testing ecosystem is a critical piece of the US public health response system. But that system is a complex tapestry of public and private institutions and actors that, in normal times, interact and influence each other indirectly or with significant time lags between action and affect. Clear coordination is an essential thread that holds that tapestry together, and it is cost-effective considering the human and financial costs of the COVID-19 pandemic. The COVID-19 pandemic, mpox emergency, and other public health challenges in recent years have shown that diagnostic testing capacity will not materialize on its own without concerted, coordinated action from the government and industry.

These recommendations are meant to inform the development of a National Diagnostics Action Plan by providing a concrete set of initial steps that can put the country on strong footing to face future pathogen threats and challenges. While we cannot know exactly what challenges lie ahead, we must prepare for future novel epidemics and pandemics. Developing and having a response plan for a strong, responsive, and adaptable national testing infrastructure is an essential piece of that preparedness.

## Declaration of Competing Interest

The authors declare the following financial interests/personal relationships which may be considered as potential competing interests: Gigi Gronvall, Sujeet Rao, and Tom Inglesby declare no conflicts of interest. Mr. Borden is a shareholder in Siemens Healthineers, and he and Susan Van Meter work for ACLA, an organization which represents clinical laboratories.

## Appendix A

• Frank Basile, M.D., Chief Executive Officer, Aegis Sciences Corporation
• Thierry Bernard President & CEO QIAGEN
• Chaeshin Chu, Ph.D Deputy Scientific Director, Director for International Affairs, Korea Disease Control and Prevention Agency
• Joel Galanter, Chief Legal Officer, Aegis Sciences Corporation
• William Morice, II, MD, PhD, President and CEO Mayo Clinic Laboratories
• Reynolds (Ren) M Salerno, PhD, Division of Laboratory Systems (DLS), Center for Surveillance, Epidemiology, and Laboratory Services, Deputy Director Public Health Scientific Services Steven Santos, PhD, Industrial Base Management & Supply Chain (IBMSC), Office of the Assistant Secretary for Preparedness and Response
• Matt Sause, CEO, Roche Diagnostics North America
• Adam Schechter Pres & CEO Labcorp
• Meg Sullivan, MD, MPH, Chief Medical Officer, Administration for Strategic Preparedness and Response, U.S. Department of Health and Human Services
• Andrea F. Wainer, Executive Vice President, Rapid and Molecular Diagnostics, Abbott.
• Jeff Wu, Deputy Director for Policy CCIIO/CMS/HHS
